# Immediate transvaginal repair of an iatrogenic full-thickness rectal injury during laparoscopic hysterectomy: a case report

**DOI:** 10.1097/MS9.0000000000005778

**Published:** 2026-10-12

**Authors:** Sindhu Para, Hayoung Wang, Jideofor Aniukwu, Matthew Mackowsky

**Affiliations:** a School of Medicine, St. George’s University, Grenada, West Indies; b General Surgery, Hudson Regional Health, NJ, USA; c General Surgery, St. Joseph’s University Medical Center, Paterson, NJ, USA

**Keywords:** gynecological surgery complications, iatrogenic rectal injury, laparoscopic hysterectomy, transvaginal repair

## Abstract

Rectal injuries are uncommon but highly morbid complications of gynecologic surgery. Prompt recognition and repair reduce the severity of complications and may yield favorable outcomes. Existing literature largely focuses on transabdominal and transanal repair techniques, which are frequently accompanied by proximal diversion with delayed reconstruction. We report a rare case of a woman who sustained a full-thickness anterior rectal wall injury during a laparoscopic hysterectomy that was repaired exclusively via a transvaginal approach during the index operation. A literature search of PubMed and Google Scholar using combinations of the terms “rectal injury,” “iatrogenic rectal injury,” “transvaginal repair,” “laparoscopic hysterectomy,” and “gynecologic surgery” identified reports describing laparoscopic, transanal, and delayed transvaginal repair techniques, but no published cases describing immediate transvaginal repair of an iatrogenic full-thickness rectal injury sustained during laparoscopic hysterectomy. This case appears to represent the first reported description of this operative approach. The authors propose that this alternative approach may prove useful in the management of similar injuries.

## Introduction

Rectal injury is a known complication during gynecological surgery, especially during procedures involving proximity to the cervix and posterior vaginal fornix. The risks of rectal injuries include sepsis, rectovaginal fistula, pelvic floor dysfunction, and the need for colostomy. Traditional management strategies for iatrogenic rectal injuries rely on transabdominal or transanal approaches for primary repair or resection. Although primary repair is increasingly favored, intraoperative management options remain limited by exposure when using laparoscopic and transanal approaches^[^1,2^]^. Here, we present a case illustrating an operative approach of immediate repair of full-thickness rectal injuries via a transvaginal route.

HIGHLIGHTS

- Rectal injury during gynecologic surgery presents significant intraoperative management challenges.
- Immediate recognition allowed for definitive management without fecal diversion or laparotomy.
- A transvaginal approach provided optimal exposure for primary repair when laparoscopic and transanal approaches were not feasible.
- Intraoperative leak testing confirmed the integrity of the repair.
- Transvaginal repair may be considered in carefully selected cases of iatrogenic rectal injury.

## Case report

A 51-year-old woman with a history of diabetes mellitus, peripheral neuropathy, and uterine fibroids presented for elective laparoscopic total hysterectomy with planned vaginal extraction. The procedure was initiated by the gynecology service. Following laparoscopic dissection, the uterus, cervix, and fallopian tubes were removed vaginally. The largest fibroid had been amputated from the uterine specimen prior to extraction.

During vaginal manipulation with the placement of a speculum, the vaginal cuff was grasped with Allis clamps, and an inadvertent full-thickness rectal injury occurred. Acute Care Surgery was consulted intraoperatively. Examination in the dorsal lithotomy position revealed a 3 × 2-inch, full-thickness anterior rectal wall injury extending from the extraperitoneal rectum into the peritoneal cavity.

Due to the extremely low position and the presence of an extraperitoneal component, laparoscopic repair was not feasible. The injury was also too proximal to be amenable to transrectal repair. Adequate visualization of the injury was achieved transvaginally using a colposcope. The rectal injury was repaired via a transvaginal approach with a single-layer, full-thickness running suture using 2-0 silk.

Rigid proctoscopy with air insufflation was used to assess repair integrity. A laparoscopic suction irrigator was used to submerge the repair during insufflation, with no evidence of an air leak. Given the absence of gross contamination, immediate repair at the index operation, and confirmation of repair integrity with an intraoperative leak test, fecal diversion was not pursued. The vaginal cuff was then closed with a running 0-Vicryl suture. The remainder of the case proceeded without further complication.

Postoperatively, the patient resumed bowel function by postoperative day 2, reporting passage of flatus and bowel movements. On postoperative day 2, the patient developed a fever and tachycardia. Blood cultures grew *Enterobacter* species, and chest radiography demonstrated a patchy left basilar opacity consistent with pneumonia. The patient was treated with intravenous piperacillin-tazobactam, with subsequent clearance of bacteremia, as confirmed by repeat blood cultures. The remainder of the postoperative course was uncomplicated, and the patient was discharged home on postoperative day 7 with a course of oral antibiotics.

At the 2-week postoperative follow-up with the obstetric team, the patient reported no fever, chills, nausea, vomiting, or other concerns. Physical examination revealed no evidence of rectocele, fistula formation, wound complications, or other postoperative abnormalities. No additional imaging or endoscopic evaluation was performed, as there were no clinical indications of postoperative complications. The patient was recovering appropriately without evidence of recurrent infection or other surgical complications. She also followed up with her primary care physician at 2 weeks and 1 month postoperatively, with no surgical complications identified at either visit.

## Discussion

The rectum extends from the rectosigmoid junction, defined by the splaying of the taeniae coli, to the dentate line of the anal canal, encompassing both intra- and extraperitoneal portions^[^3^]^. The posterior vaginal wall lies immediately anterior to it, separated by the rectovaginal fascia, also known as the fascia of Otto. Histological examination demonstrates minimal fascial separation between the muscular layers of the posterior vaginal wall and the anterior rectal wall, underscoring the susceptibility of the rectum to injury during vaginal and pelvic procedures^[^3^]^.

Management principles for rectal injuries have largely been extrapolated from the trauma literature. Non-destructive intraperitoneal injuries are treated with trans-abdominal repair, while destructive injuries are treated with resection, with or without diversion^[^4–6^]^. Historically, extraperitoneal injuries were managed with primary repair, pre-sacral drainage, rectal washout, and proximal diversion^[^6,7,8^]^. Contemporary guidelines increasingly favor primary repair without routine diversion for accessible, non-destructive injuries^[^6,7^]^.

Despite evolving recommendations, guidance on the immediate intraoperative management of iatrogenic rectal injuries occurring during gynecologic surgery remains limited. A literature search identified reports describing laparoscopic repair of rectal injuries as well as elective transvaginal repairs, including fistula, rectocele, prolapse, or cuff dissection procedures^[^9–11^]^. Transvaginal repair has also been described for low rectovaginal septal injuries sustained during childbirth within hours of the injury^[^12^]^.

The present case is unique in that the transvaginal approach using a natural orifice provided excellent exposure of a defect that was not amenable to either laparoscopic or transrectal repair, allowing definitive closure without laparotomy or fecal diversion. Successful management was likely facilitated by several favorable factors, including immediate intraoperative recognition, a non-destructive full-thickness injury, minimal contamination, adequate transvaginal visualization, and confirmation of repair integrity with intraoperative leak testing. Together, these factors permitted safe primary repair during the index operation while avoiding the morbidity associated with more invasive approaches. In the absence of these factors, traditional approaches –including transabdominal or transanal repair with or without fecal diversion –remain appropriate. Because this is a single case report, the findings are inherently limited in generalizability and cannot establish the safety, reproducibility, or superiority of this technique compared with conventional approaches. Successful application of this strategy likely depends on careful patient selection, including immediate intraoperative recognition, favorable anatomy, minimal contamination, adequate transvaginal exposure, and confirmation of repair integrity. Additional case reports and larger studies are needed to better define appropriate patient selection, technical considerations, and long-term outcomes before this approach can be recommended more broadly. Nevertheless, awareness of this option may expand the armamentarium for managing iatrogenic rectal injuries in appropriately selected cases.

## Conclusion

Immediate recognition and transvaginal repair of a full-thickness rectal injury can result in successful primary closure without the need for diversion or abdominal intervention. In patients with limited contamination and favorable exposure, a transvaginal approach may provide a safe and effective alternative to traditional laparoscopic, transanal, or open repair strategies. Reporting such cases contributes to the growing body of literature informing the management of rectal injuries during gynecologic surgery.

## Data Availability

Data sharing not applicable to this article as no datasets were generated or analyzed during the current study.

## Additional information

The author(s) declared no potential conflicts of interest with respect to the research, authorship, and/or publication of this article.

## Statement of non-duplication

All authors certify that this manuscript is a unique submission and is not being considered for publication by any other source in any medium. The manuscript has not been published, in part or in full, in any form.
